# The patient-reported outcome measure for older people living with frailty receiving acute care (PROM-OPAC): field-testing and validation

**DOI:** 10.1186/s41687-024-00796-8

**Published:** 2024-10-16

**Authors:** James D. van Oppen, Simon P. Conroy, Jagruti Lalseta, Nicola Mackintosh, Peter Riley, Vivien Richardson, Jose M. Valderas, Timothy J. Coats

**Affiliations:** 1 Centre for Urgent and Emergency Care Research, University of Sheffield, Sheffield, United Kingdom; 2 College of Life Sciences, George Davies Centre, University of Leicester, Leicester, UK; 3 MRC Unit for Lifelong Health and Ageing, University College London, London, UK; 4 Leicester, Leicestershire and Rutland Older Persons Patient and Public Involvement Forum, Leicester, UK; 5 Department of Family Medicine, National University Health System, Singapore, Singapore; 6 Emergency and Specialist Medicine, University Hospitals of Leicester NHS Trust, Leicester, UK

**Keywords:** Frailty, Emergency care, Patient-reported outcome measure

## Abstract

**Background:**

Current acute healthcare service metrics are not meaningful for older people living with frailty. Healthcare knowledge, situational security, and physical and psychosocial function are important outcomes typically not collected. The use of patient-reported outcome measures (PROMs) could support these assessments. Existing instruments are not comprehensive as they typically consider function, while older people with frailty also value enablement (self-determination and security in health and healthcare). This study field-tested and validated a PROM for older people with frailty receiving acute care (PROM-OPAC) to measure enablement.

**Methods:**

People aged 65+ with Clinical Frailty Scale 5–8 were recruited within seventy-two hours of an emergency attendance. Iterations of the novel instrument were administered over three stages: (1) preliminary field-testing for reliability (response distribution and internal consistency) and structure (exploratory factor analysis, EFA); (2) intermediate field-testing of an improved instrument for reliability and structure; (3) final draft validation assessing reliability, structure (confirmatory factor analysis, CFA), and construct validity based on a priori hypotheses. Feasibility was appraised throughout using data completeness and response rates and times.

**Results:**

241 people participated. Three items of a preliminary seven-item measure had poor response distribution or loading and were accordingly improved. The intermediate instrument had interpretability issues and three items required further improvement. The final eight-item draft had acceptable reliability (Cronbach’s alpha: 0.71), structure (two factors for self-determination and security; RMSEA: 0.065; TLI: 0.917; CFI: 0.944), and construct validity (lower scores from respondents waiting longer and requiring admission). Feasibility was promising (response rate 39%; 98% responses complete; median completion time 11 (IQR: 12) minutes).

**Conclusions:**

Administration of the PROM-OPAC appeared feasible and the instrument had acceptable psychometric properties. Further evaluation is required to assess generalisability.

## Background

Health systems typically appraise acute healthcare for quality using service metrics such as waiting times, hospital admissions and reattendances, and mortality. These have limited relevance for older people living with frailty, who have more complex healthcare needs and particularly benefit from a person-centred approach [1, 2]. Acute frailty care should be framed around comprehensive geriatric assessment, a person-centred model of care but which has rarely been appraised using person-centred measures of outcome [3]. More meaningful markers of healthcare effectiveness for this population would capture outcome goals related to the person’s health-related quality of life (HRQoL) [4, 5]. Measurement of these might encourage and stimulate the configuration of healthcare processes around attaining outcomes that are meaningful [2].

Such outcomes cannot be measured using timestamp-derived service metrics but instead require self-reporting. This can be achieved using patient-reported outcome measures (PROMs)—questionnaire-based instruments with psychometric reliability and validity. PROMs data have applications throughout healthcare for people living with frailty, at the micro (aiding elicitation and communication of healthcare goals), meso (evaluating service delivery), and macro (commissioning programmes) levels [6].

For research and clinical purposes PROMs have been collected in hospital from older people living with frailty. Existing instruments are suited to this population’s goals to maintain or recover physical and psychosocial functioning (COOP/WONCA, EuroQol EQ-5D, McGill Quality of Life Questionnaire, Palliative care Outcome Scale [7–10]), with the EuroQol perhaps presenting a feasible compromise between instrument brevity and meaningful coverage of function outcomes [11]. However, these PROMs do not comprehensively consider all acute healthcare outcome goals among older people living with frailty, which in addition to function, typically and perhaps uniquely also include themes such as security of health and certainty of situation [12, 13]. Previously described as autonomy with the sub-themes information, control, and security, conceptual indistinction between the former labels might be resolved by their merging as *self-determination* encompassing participation, competence, and relatedness [14, 15]. Self-determination and security here represent the capabilities to achieve *enablement* [16–18]. Consideration of enablement is warranted with older people living with frailty given the uncertainty and vulnerability experienced typically and perhaps uniquely by this population during acute healthcare.

Therefore, a preliminary PROM for older people living with frailty receiving acute care (‘PROM-OPAC’) was developed from underpinning qualitative evidence reflecting healthcare knowledge, involvement in decision-making, and situational security [13]. Co-creation and cognitive pre-testing have been described previously [19]. The resulting preliminary items considering enablement are tested further in this current study.

This paper describes field-testing and validation of the PROM-OPAC. The objectives of this study were to (1) field-test and improve the preliminary instrument in older people living with frailty receiving acute care, and (2) examine the final draft instrument for metric performance, including construct validity.

## Methods

The study tested iterative instrument drafts with older people receiving acute care and modified these for improvement using feedback from participants. The project engaged extensively with three lay research partners in ongoing co-creation, as described previously [19]. Their major role was to review items ‘flagged’ as potentially problematic in analyses for modification, discarding, or replacement, and in doing so assured that materials were easily accessible by the intended audience.

The study was conducted in three stages: preliminary field-testing and improvement, intermediate field-testing which was terminated early to further improve a problematic item, and finally validation. Feasibility of PROMs collection was appraised throughout using times and completeness of responses.

### Settings and participants

Acute hospitals were identified through the UK Clinical Research Network to represent a variety of regions and care process models. The eight sites included small to large regional hospitals (650–938 beds) and a Major Trauma Centre (1700 beds).

Older people living with frailty and receiving acute healthcare were identified by research practitioners. They were aged 65+ and were living with mild to very severe frailty, defined as score 5–8 on the Clinical Frailty Scale, administered by clinical teams [20]. Potential participants were recruited in hospital within seventy-two hours of unscheduled attendance. This timeframe for acute care was selected as the period of diagnostic and prognostic uncertainty associated with multiple care transitions and interventions, during which time participants were receiving care in emergency departments, acute assessment areas, and acute care wards. Printed information was given and explained. People were not excluded based on presenting or co-morbid conditions but did need to provide consent. This was indicated personally and formalised in writing either by participants themselves or by consultees where participants had capacity to respond but not consent. For example, a person living with cognitive impairment may have been able to agree with research aims and respond to the instrument, but not to process the information leaflet or sign consent.

Each stage recruited five to ten participants per novel item, following available guidance and literature [21, 22]. The study was approved by an NHS research ethics committee and the Health Research Authority (ref 21/WM/0049).

### Administration of instrument(s)

Participants were invited to complete PROMs during natural waiting periods between assessments, treatments, or transfers in the acute setting. They responded with or without reading and scribing assistance from the person accompanying them or the research practitioner, according to their preference and needs. Where there were barriers to communication (such as stroke), the person accompanying was able to support expression but not to respond on participants’ behalf. In this development stage, the research practitioner collected participants’ own responses; there were no responses by proxy.

All participants completed a version of the PROM-OPAC during the initial visit. The PROM-OPAC used a consistent five-level ordinal scale: agree strongly, agree, neither agree nor disagree, disagree, disagree strongly. The version for each participant depended on the stage in which they were enrolled. The EQ-5D-5L, EQ-VAS, and single-item Self Rated Health were also collected and are reported separately [11]. The research practitioner recorded participants’ age, Clinical Frailty Scale, self-reported gender, and self-reported ethnic group using Office for National Statistics categories.

PROM-OPAC items were scored 5 (agree strongly) to 1 (disagree strongly) so that higher total scores represented better agreement with the statements. To account for potential non-linearity of response levels, scores were also calculated as participants’ affirmation or their proportion of responses at ‘agree’ and ‘agree strongly’. Using this approach, higher scores represented better fulfilment of the considered outcomes.

The preliminary field-testing stage examined the instrument for issues with reliability and structure. Response rate during this preliminary stage was calculated, using screening logs for people approached for consent. Preliminary stage participants were invited to return a retest within one month of being discharged. This was sent to their home in their choice of electronic or paper format. Format preference was recorded. The retest was identical except for an additional five-level health change item anchored ‘much better’ to ‘much worse’. Problematic preliminary items were flagged and improved through lay review and cognitive testing. Cognitive testing was with patient participants in the target demographic group, sampled purposively with reparative intent (to identify and correct problematic statements or interpretation) as previously described [19]. This improved instrument was then administered in the intermediate field-testing stage, which again sought to identify and improve issues with reliability and structure. Finally, the validation stage administered and examined the draft PROM-OPAC for reliability, structure, and construct validity [23]. In all stages, feedback for improvement was collected with a modified Likert scale for relevance, ease of completion, and appearance (5 levels, very good to very poor) and an optional white-space field.

### Analyses

Demographic characteristics were summarised to assess adequacy of recruitment. Feasibility was evaluated using response rates for the preliminary stage, and with the full dataset across all three study stages, the median and range for completion time were calculated as markers of participant and practitioner burden and the proportion of missing responses were presented overall and for each item. Qualitative feedback and recommendations were analysed using simple content analysis for positive and negative attitudes [24, 25]. Quantitative analyses were performed using R Studio with packages *ggplot2*,* lavaan*,* mice*, and *psych* [26–28].

#### Preliminary field-testing

Reliability assessment used response distribution (per-item response levels, end effects, and standard deviation) and internal consistency (Cronbach’s alpha statistic) [23]. Item standard deviation > 0.95 and alpha > 0.70 were considered acceptable. Structure assessment used exploratory factor analysis (EFA), appraising the characteristics Root Mean Square Error of Approximation (RMSEA, excellent < 0.06, poor > 0.10), Tucker-Lewis Index (TLI, excellent > 0.95, poor < 0.90), and Comparative Fit Index (CFI, excellent > 0.95, poor < 0.90) [29].

Participants missing > 15% responses were excluded from EFA and remaining missing data were replaced with imputation. Data were first screened for factorability using Bartlett’s and Kaiser-Meyer-Olkin tests, and normality, linearity, and homogeneity of residuals were screened for satisfaction of factor analysis assumptions. The number of factors was estimated using eigenvalue scree plots, parallel analysis, and theoretical derivation. Models were produced iteratively and compared for two and three factor solutions, using maximum likelihood and diagonally weighted estimations and with trial exclusion of items. Those best-performing used diagonally weighted least squares mathematics (appropriate for the ordinal scale) with direct oblique rotation (allowing correlation between the related autonomy sub-themes) [30].

Items with end-effects, poor distribution, poor inter-relatedness, or unexpected factor loading were flagged for review with lay research partners. Retest data were analysed using the Wilcoxon Signed Rank Test for paired PROM-OPAC measurements and ANOVA to compare health change with PROM-OPAC change.

Flagged items were iteratively rephrased, amended, and appended collaboratively with lay research partners. All substantial modifications were evaluated for content validity and interpretability in reparative cognitive interviews with patient participants, as described in previous work [19].

#### Intermediate field-testing

The resulting intermediate instrument was administered to a new cohort of patient participants receiving acute hospital care. Responses were assessed for reliability and structure as in the preliminary stage.

Interim analysis showed that further improvement of problematic items would be necessary. Recruitment to this intermediate stage was therefore terminated early. Items with poor distribution or loading were again flagged for review and amended with lay research partners. Modifications were again appraised with patient participants using cognitive interviews.

#### Validation

The improved draft was administered to a final cohort of participants. Responses were analysed for reliability as in previous stages and for structure using confirmatory factor analysis (CFA). For construct validity, we assessed hypotheses for lower PROM-OPAC scores with increasing age, frailty, poorer ED process outcomes (waiting times and admission), and worse thirty-day service outcomes (longer admissions, reattendances, and mortality) using correlation and Kruskal-Wallis tests. Iterative CFA models were then compared with trial removal of items. The resulting simplified solutions with acceptable performance were re-examined for construct validity.

## Results

### Recruitment

241 participants were recruited across the three stages (Table 1). Their age (median: 85; range: 65–102), frailty (median CFS: 6; range: 5–8), and gender (61% female) represented the UK population of older people living with frailty. Recruitment of people with non-white ethnic group was very low (1%).

**Table 1:** Characteristics of participants recruited to the PROM administration stages

	Total	Summary by-study stage (*n*)		
		Preliminary field-testing	Intermediate field-testing	Final validation
Recruiting sites	**8 (A–H)**	A, B, C	C, D, E	F, G, H
Participants, *N*	**241**	128	47	66
Female, %	**60.9**	61.7	48.9	68.2
Non-white ethnic group, %	**1.0**	1.6	2.1	1.5
Age/years, median (range)	**85 (65–102)**	85 (65–102)	85 (72–97)	86 (67–97)
CFS, median (range)	**6 (5–8)**	6 (5–8)	6 (5–7)	6 (5–7)

*CFS* clinical frailty scale

Of people approached for recruitment during the preliminary stage, 39% participated and responded. Data completeness was high, with 98% participants across the three stages responding to all items. Median time for PROM completion was 11 (IQR: 12) minutes. Research practitioners reflected that for some participants the questions prompted conversation and discussion about health, healthcare, and outcome goals, and therefore completion took longer.

Participants were complimentary about the instrument’s relevance (79% very good or good), reporting that completing the items had helped them to reflect on their situation. The topics were clear to navigate. The holistic focus was appreciated, but comments were noted from some who would have preferred enquiry to be more specific to their presenting problem. While the instrument was judged easy to complete (92% very good or good), participants were divided between wanting a shorter instrument and wanting additional questions. Suggestions included presenting comments boxes with each item to allow better communication of perspectives. Feedback on the preliminary instrument appearance (76% very good or good) informed accessibility improvements with larger font size and lowercase.

### Preliminary field-testing

Seven items were administered. Ceiling effects were observed with items D2 (“I know how serious my problem is”) and D6 (“I have enough support where I live”), where 30 and 41% participants respectively agreed strongly (Table 2, column 3). The suboptimal response distribution (SD, 0.95) for item D4 (“professionals listen to my choices about healthcare”) was flagged as perhaps indicating reluctance to respond negatively about clinicians. Internal consistency was adequate (alpha: 0.73; 95% CI: 0.66–0.80) and did not improve with removing any items (Table 2, column 4).

No participants’ responses were excluded from EFA for missing data, and maximum 1.56% (median, 0.78) responses per item required imputation. EFA models had excellent fit statistics for both two (RMSEA: 0.000; 95%CI: 0.000–0.078; TLI: 1.000; CFI: 1.000) and three-factor solutions (RMSEA: 0.000; 95%CI: 0.000–0.095; TLI: 1.000; CFI: 1.000). As parallel analysis suggested two factors, these item loadings are presented (Table 2, column 5): D4 loaded poorly (0.392) onto factor 1 and was again flagged for review.

**Table 2:** Reliability and structural properties of the preliminary instrument

Item	Reliability			Structure
	Distribution (SD)	Distribution (Ceiling response)	Internal consistency (Alpha if item removed)	EFA (Factor with loading > 0.4)
D1: I know the results of my tests and investigations	1.23	0.21	0.68	1
D2: I know how serious my problem is	1.15	***0.30***	0.70	1
D3: I know what happens next with my healthcare	1.25	0.18	0.66	1
D4: Professionals listen to my choices about healthcare	***0.95***	0.21	0.70	***NA***
D5: I feel in control of my life	1.21	0.28	0.70	2
D6: I have enough support where I live	1.18	***0.41***	0.73	2
D7: I feel safe living with my health problems	1.04	0.21	0.72	2

Items with potential issues, shown highlighted, were flagged for review. Overall alpha: 0.73

**Fig. 1:**
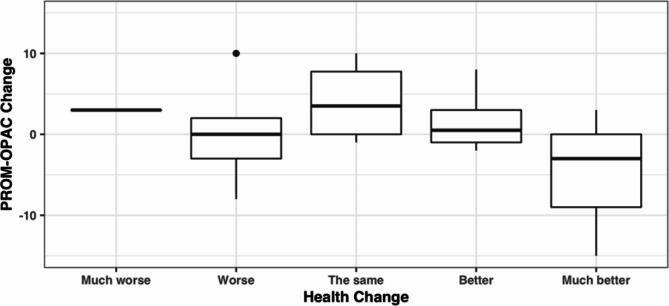
Change in PROM-OPAC total score with responses to the health change item (33 respondents representing 26% retest response rate)

103 (86%) participants opted for paper format retest. The retest response rate was 26%, with no difference between formats. Median total PROM-OPAC scores did not significantly change at retest (Wilcoxon *p* = 0.248). There was a non-significant relationship (ANOVA *p* = 0.277) between health change and PROM-OPAC change at retest (Fig. 1).

With lay research partners, the problematic item D4 (professionals listen to my choices about healthcare) was modified to maintain consistency of subject, becoming E4 ‘I feel listened to in decisions about my healthcare’. D5 was amended to clarify the focus of control as being over one’s health. Two new items were introduced (Table 3, Action 1), yielding nine items for the next stage of testing. These alterations were appraised in five cognitive interviews, with recruitment from those participants had recently completed the instrument at Site A. Their age ranged from seventy-two to eighty-five and they were living with mild to severe frailty (CFS 5–7). Three participants were female and two were male.

**Table 3:** Instrument improvements during the intermediate analyses

ID1	Preliminary item stem	Action 1	ID2	Intermediate item stem	Action 2	ID3	Validation draft item stem
D1	I know the results of my tests and investigations	Keep	E1	I know the results of my tests and investigations	Keep	F1	I know the results of my tests and investigations
D2	I know how serious my problem is	Keep	E2	I know how serious my problem is	Keep	F2	I know how serious my problem is
D3	I know what happens next with my healthcare	Keep	E3	I know what happens next with my healthcare	Keep	F3	I know what happens next with my healthcare
D4	Professionals listen to my choices about healthcare	Modify	E4	I feel listened to in decisions about my healthcare	Modify	F4	I am able to influence decisions about my healthcare
D5	I feel in control of my life	Modify	E5	I feel in control when managing my health problems	Keep	F5	I feel in control when managing my health problems
–	–	–	E6	I feel in control about involving other people in my healthcare	Modify	F6	I can choose who else is involved in my healthcare
D6	I have enough support where I live	Keep	E7	I have enough support where I live	Keep	F7	I have enough support where I live
D7	I feel safe living with my health problems	Keep	E8	I feel safe living with my health problems	Keep	F8	I feel safe living with my health problems
–	–	–	E9	I feel safe to continue with my present living arrangements	Modify	F9	I feel safe with my present living arrangements

### Intermediate field-testing

Interim analysis of responses to nine intermediate field-testing items identified poor distribution for items E4, E5, and E8 (SD, 0.73, 0.85, and 0.84), suggesting ongoing issues with interpretation and requirement for further improvement (Table 4). Additionally, item E6 caused confusion with unclear phrasing. Addition of the two new items improved overall internal consistency to 0.81 (95% CI: 0.71–0.88). EFA models again had excellent characteristics for both two- and three-factor solutions (RMSEA: 0.000; 95%CI: 0.000–0.000; TLI: 1.000; CFI: 1.000), with parallel analysis again suggesting two factors. In that model, however, item E6 did not load onto either factor.

**Table 4:** Reliability and structural properties of the intermediate instrument

Item	Reliability			Structure
	Distribution (SD)	Distribution (Ceiling response)	Internal consistency (Alpha if item removed)	EFA (Factor with loading > 0.4)
E1: I know the results of my tests and investigations	1.00	0.11	0.79	1
E2: I know how serious my problem is	1.11	0.23	0.80	1
E3: I know what happens next with my healthcare	1.04	0.11	0.77	1
E4: I feel listened to in decisions about my healthcare	***0.73***	0.15	0.78	1
E5: I feel in control when managing my health problems	***0.85***	0.09	0.80	2
E6: I feel in control about involving other people in my healthcare	1.01	0.30	0.81	***NA***
E7: I have enough support where I live	1.16	0.26	0.77	2
E8: I feel safe living with my health problems	***0.84***	0.15	0.79	2
E9: I feel safe to continue with my present living arrangements	1.02	0.28	0.78	2

Problematic items were again flagged for review. Overall alpha: 0.81

Item E6 was amended to F6 ‘I can choose who else is involved in my healthcare’ (Table 3, Action 2). E4 was adjusted to reflect active rather than passive involvement in decision-making, and E9 was shortened to consider the living setting rather than the decision to return there. These modifications were examined for interpretability during six further cognitive interviews. These participants had not previously completed the instrument. Their age ranged from seventy-four to ninety-five and they were living with mild to severe frailty (CFS 5–7). Four participants were female and two were male. Five participants were living in their own homes, and one was living in residential care.

### Validation

There were no ceiling effects with the nine-item validation draft instrument. Overall internal consistency was acceptable (alpha: 0.74; 95%CI: 0.64–0.83) and did not improve with removal of any item. Response distribution was inadequate for item F6 (0.67) and poor for F9 (0.90) (Table 5). There were no missing data in this phase. Confirmatory factor analysis with two dimensions yielded excellent model characteristics (RMSEA: 0.034; 95%CI: 0.000–0.104; TLI: 0.980; CFI: 0.986).

**Table 5:** Reliability and structural properties of the validation draft instrument

Item	Reliability			Structure
	Distribution (SD)	Distribution (Ceiling response)	Internal consistency (Alpha if item removed)	CFA (Factor: loading)
F1: I know the results of my tests and investigations	1.16	0.17	0.73	1: 0.55
F2: I know how serious my problem is	0.95	0.27	0.72	2: 0.46
F3: I know what happens next with my healthcare	1.14	0.14	0.72	1: 0.64
F4: I am able to influence decisions about my healthcare	1.10	0.19	0.71	1: 0.76
F5: I feel in control when managing my health problems	1.03	0.16	0.70	1: 0.68
F6: I can choose who else is involved in my healthcare	***0.67***	0.22	0.71	1: 0.65
F7: I have enough support where I live	1.05	0.27	0.74	2: 0.57
F8: I feel safe living with my health problems	1.01	0.19	0.73	2: 0.65
F9: I feel safe with my present living arrangements	***0.90***	0.29	0.73	2: 0.59

PROM-OPAC was not associated with increasing age (Spearman *p* = 0.609) or frailty (Kruskal-Wallis *p* = 0.240). Lower PROM-OPAC scores were observed in respondents with longer waits at participation (Spearman *p* = 0.019) and who required admission to hospital (Kruskal-Wallis *p* = 0.002). Those with lower PROM-OPAC had longer subsequent hospital admission (Spearman *p* = 0.025), but similar reattendances (Kruskal-Wallis *p* = 0.241) and deaths (Kruskal-Wallis *p* = 0.102).

**Fig. 2:**
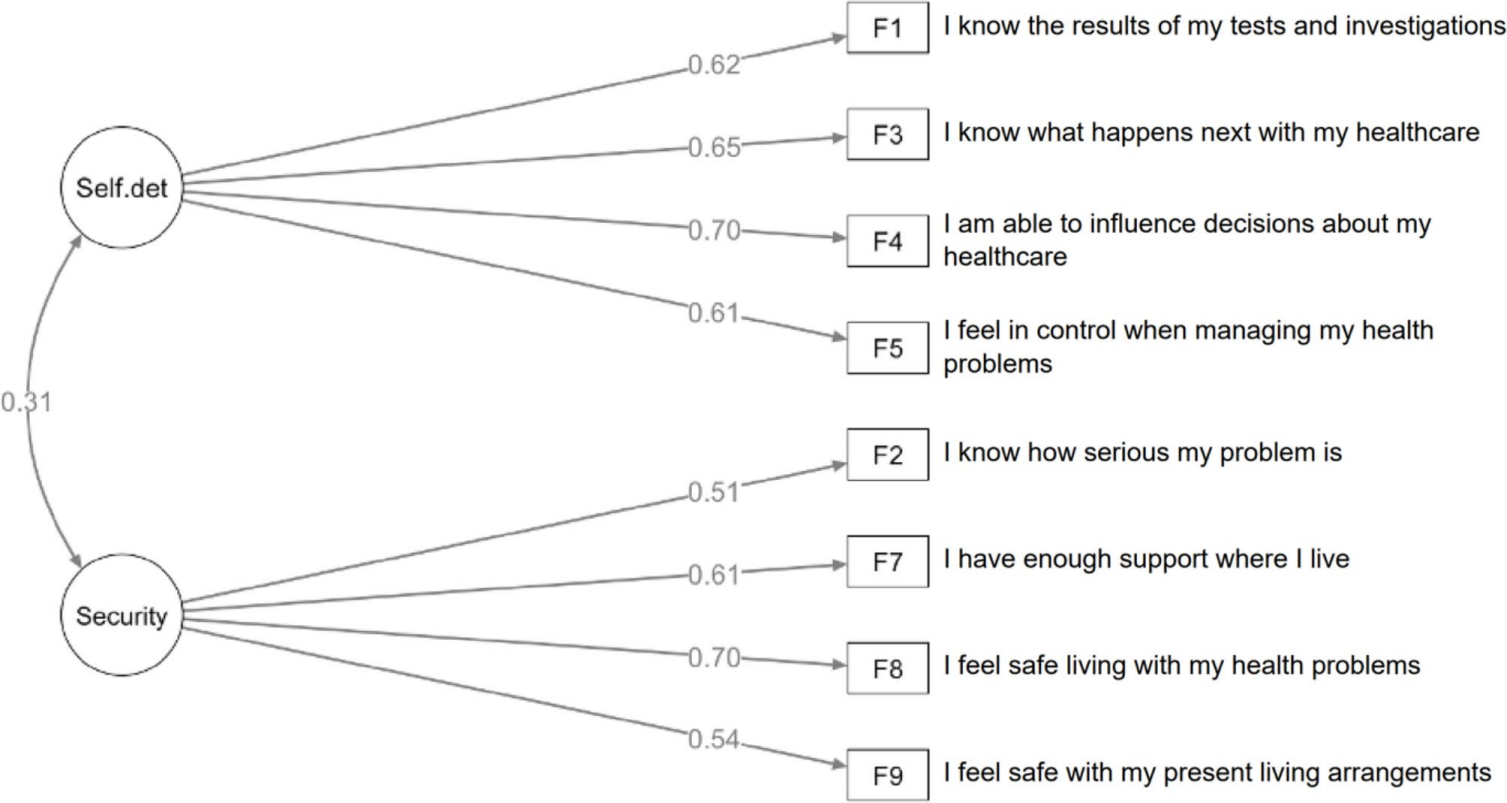
Conceptual structure of the final eight-item PROM-OPAC instrument to measure enablement and its sub-themes self-determination and security. *Self.det* self-determination

A final two-factor model with trial removal of one item with poor response distribution suggesting an issue with interpretation (F6) had acceptable fit statistics (RMSEA: 0.065; 95% CI: 0.000-0.132; TLI: 0.917; CFI: 0.944 (Fig. 2)) and adequate internal consistency (alpha: 0.71; 95% CI: 0.59–0.81). The results of construct validation analyses were unchanged using this simplified eight-item solution.

## Discussion

### Summary of findings

Administration of the draft PROM-OPAC appeared feasible, with 39% initial response rate, most participants requiring under fifteen minutes to respond, and with excellent (98%) completeness. Participants reported positively on item relevance and overall ease of completion. The final eight-item PROM-OPAC had acceptable reliability and structure and had construct validity with hypotheses for waiting times and hospital admissions. The PROM-OPAC construct did not correlate with ED reattendances or mortality, and may therefore contribute an additional dimension in considering outcomes of acute frailty care.

### Limitations

While the participants had age and frailty ranging representatively across the older continuum, the study recruitment failed to represent diversity of ethnicity. Only 1.6% participants had non-white ethnicity categories when around 20% was expected, so the results may not be generalisable to ethnically diverse populations [31]. This finding is, regrettably, consistent with medical literature frequently failing to recruit ethnically representative populations [32]. Recruitment was reflected upon with research staff and learning points have been shared [33, 34]. Social factors may have influenced participation, as recruiting research practitioners were mostly white [32]. Language was identified as a contributing factor as at the time of study the instrument was available only in English. While samples were sized based on consensus recommendations, the modest final validation cohort is acknowledged, and further external validation of these findings is warranted.

Participants completing the instrument with a research practitioner present prompts consideration of acquiescence bias, as respondents may have felt unable to report negatively on their healthcare [35]. However, participants gave feedback that having the researcher present was beneficial and aided reflection. Similar reflections emerge around the presence of an accompanying person, with abuse of older people being frequently unrecognised [36]. The potential for bias requires cautious balance with the potential for improved accessibility. The timing and setting may also have influenced responses as participants were likely to have felt tired and unwell during initial involvement [37]. At this stage of development, such factors were not further explored among participants completing the identical retest. The change in participants’ reported health state following acute care precluded assessment of retest reliability, and such evaluation would require an alternative study design. There were minimal missing data; however, participants were not consecutively recruited and over-representation of individuals who were able to self-report the PROM may have led to selection bias. The feasibility findings reported in this study are limited to data collected in the context of research, and further evaluation is necessary during implementation into normal care processes.

While the concept elicitation study purposively recruited people living with dementia, this current psychometric work recruited older people with frailty generically and did not collect data on cognitive impairment or other contributing factors such as socioeconomic status and literacy. Around one third of older people using the emergency department might be expected to have a dementia or delirium [38]. Further work is required to determine feasibility of PROMs participation with population sub-groups.

Departmental records of attendance times and service outcomes were used as construct validators, representing the mainstay of UK health service metrics. Participants were recruited to this study during 2021-23, when NHS time-based performance was at its poorest ever. Media attention plausibly could have influenced participants’ expectations for their attendance and subsequent satisfaction. While construct validators were selected acknowledging the absence of a gold standard measure for acute care autonomy, the meaningfulness of time targets in the current context appeared particularly limited.

Lay research partners did not receive specific training for their role in selection or improvement of measure items; while this may have encouraged reinforcement that their experiential knowledge was valued equally with researchers’ technical assessment, an effect on confidence to contribute and thus range of portrayed perspectives warrant consideration [39]. Some imbalances of decision-making power will have persisted as the researcher assumed final responsibility for actions.

### Relationships with existing knowledge

Data completeness here was excellent—98% complete compared to 90% in studies with older people in other settings [40]. This was with research practitioners present at administration, although there were no missing data among the retest responses from unaccompanied participants during the initial field-testing cohort. The retest response rate for those participants was far lower (26%) than that achieved in longitudinal assessment of people with mild cognitive impairment (89%) [41], but in keeping with recent urgent and emergency care surveys ranging 14–30% [42–44]. Those researchers observed improved response when telephone or email reminders were employed. The acute care setting is characterised by uncertainty and illness severity, which may explain poorer retest response here had participants felt too unwell to post their retest. Their perspectives on healthcare and research participation may also have changed during the follow-up period, and further work is warranted to evaluate methods for collecting PROMs after acute care discharge. The time and support required for instrument collection, while feasible with funded research practitioner support, may restrict real-world PROM administration in busy acute care settings to those participants able to self-report unaided. Professional accompaniment may have inflated data completeness but was noted to have prompted (desirable) discussions and reflections with participants about outcome goals. The timing and mechanisms for professionally supported administration outside of research applications will require consideration.

Only a minority (6%) of participants expressed a preference for electronic retest format. This was at odds with knowledge of older people’s use of the Internet [45], and may represent an cohort effect given that we focused on recruiting participants living with frailty. Around half of participants with frailty were expected to have been able to complete an electronic instrument independently [46]. Some postal respondents may have been Internet users but preferred the convenience of paper over learning to use a new system [47]. Being acutely unwell may also have influenced preference, as previously people with poorer self-reported health have required paper alternatives to web surveys [48].

Contrary to our hypotheses, the PROM-OPAC scores did not decrease with increasing age or frailty. A study of a generic HRQoL PROM with longitudinal frailty data observed people who were older to more often overestimate their health (“health optimistic”) [49]. This was explained as being related to response shift. Elsewhere, a reinterpretation of ontology describes frailty as altered ability to affect and be affected [50]. Thus, people’s preferences and expectations for healthcare self-determination may have shifted with frailty progression: while responses appeared to hold metric validity in the present, their suitability for sequential observation requires further investigation. The finding also prompts reconsideration of assumptions regarding change in outcome goals among older people, as these have been shown not to vary with increasing frailty [51].

There is growing recognition that current acute and emergency care metrics lack comprehensiveness and meaningfulness, and a suite of patient-reported measures have recently been developed: the PRM-Acute Care, PROM-ED, PREM-ED 65+, and PROM-OPAC [52–54]. In common these capture the uncertainty of acute illness and recovery with consideration of healthcare information and decision-making. The security domain is unique to the PROM-OPAC and represents the vulnerability associated with living with frailty. Health system leaders and commissioners should appraise available measures for their relevance and comprehensiveness to their own service, while future collaborative work might seek to cross-culturally validate, map, and combine these instruments for efficient and compatible deployment.

## Conclusions

PROM-OPAC is an eight-item measure of enablement (self-determination and security in health and healthcare) for older people living with frailty receiving acute care. It appeared feasible to administer in a research application and had acceptable properties for response distribution, internal consistency, structure, and construct validity. Further evaluation is required with specific population groups and to establish the feasibility of routine implementation in clinical care.

## Data Availability

The datasets used and/or analysed during the current study are available from the corresponding author on reasonable request.
